# Review of Cracked Tooth Syndrome: Etiology, Diagnosis, Management, and Prevention

**DOI:** 10.1155/2021/3788660

**Published:** 2021-12-15

**Authors:** Fei Li, Yaoyao Diao, Jiayin Wang, Xingyu Hou, Shuzhan Qiao, Jiawen Kong, Yunhan Sun, Eui-Seok Lee, Heng Bo Jiang

**Affiliations:** ^1^The Conversationalist Club School of Stomatology, Shandong First Medical University and Shandong Academy of Medical Sciences, Tai'an, Shandong 271016, China; ^2^Department of Oral and Maxillofacial Surgery, Graduate School of Clinical Dentistry, Korea University, Seoul 08308, Republic of Korea

## Abstract

Cracked tooth syndrome refers to a series of symptoms caused by cracked teeth. This article reviews the current literature on cracked tooth syndrome from four aspects, etiology, diagnosis, management, and prevention, to provide readers integrated information about this. The article begins with an introduction to the odontiatrogenic factors and then covers the noniatrogenic factors that induce cracked tooth syndrome. While the former discusses inappropriate root canal therapy and improper restorative procedures, the latter covers the topics such as the developmental and functional status of cracked tooth syndrome. This is then followed by the description of common clinical diagnosis methods, the prospects of new technologies, and summaries of current clinical management methods, including immediate management and direct and indirect restoration. In the final section, preventive methods and their importance are proposed, with the aim of educating the common population.

## 1. Introduction

**Figure 1:**
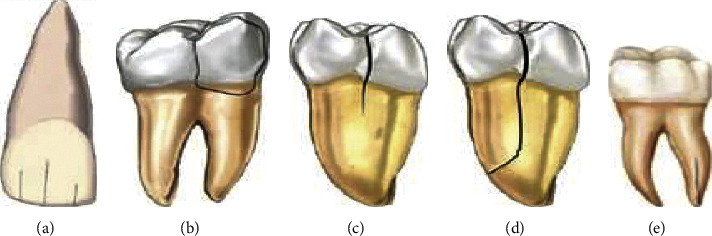
(a) Visible fracture lines within the enamel suggestive of craze lines; (b) fractured cusp terminating in the cervical part of the tooth; (c) cracked tooth extending from the occlusal tooth surface without separation of tooth fragments; (d) separated tooth fragments suggestive of a split tooth; and (e) vertical root fracture [4].

Cameron proposed [1] “cracked tooth syndrome (CTS)” in 1964 and defined it as a critical posterior tooth incomplete fracture that includes the dentin extending to the pulp. This definition was later modified by Ellis [2] as “a fracture plane of unknown depth and direction passing through the tooth structure that, if not already involved, may progress to communicate with the pulp and/or periodontal ligament.” The five most commonly used classifications of cracked teeth were provided by the American Association of Endodontists [3]. These are craze line (asymptomatic) and fractured cusp (mild pain mostly during biting and in the cold) with a better prognosis, cracked tooth (severe pain during biting) depending on the depth and extent of the fracture, split tooth (pain during chewing), and vertical root fractures (severe pain) with a poor prognosis [4] (Figure 1).

Multiple factors such as bite force and thermal cycling can cause crack lines on the tooth or damage the tooth structure [5]. The distribution of cracks is mainly in the mesiodistal region, which can be seen only on the occlusal surface and edge ridge of the crown or can be observed as an extension on the adjacent surface or even the subgingival region. The direction and depth of the fracture surface are normally difficult to predict [6]. The symptoms of CTS include spontaneous pain, bite pain, and cold irritation pain; the bite pain gets worse when the bite force increases [7]. In such situations, patients often complain and often seek medical attention. However, some research studies indicate that there is no obvious correlation between these symptoms and the appearance of cracked teeth and that not all broken teeth present with symptoms [8]. Thus, it is essential to comprehensively and meticulously diagnose CTS using auxiliary tools. There are various management methods for CTS, and statistical differences exist in the choice of methods between the specialist groups and the general practitioners [9]. Failure to diagnose and manage CTS in a reasonable manner may result in irreversible severity in symptoms, as the fracture progresses due to bacteria invading the dentin and even reaching the pulp [10].

This article reviews the literature related to the diagnosis and management of cracked teeth and provides alternative concepts in the description, etiology, diagnosis, and treatment of cracked teeth. A search in the PubMed, Embase, and Medline databases was carried out using various keywords related to cracked teeth such as “cracked tooth,” “cracked tooth syndrome,” “cracked tooth diagnosis,” “cracked tooth treatment,” “cracked tooth management,” and “cracked tooth risk factors,” and other keywords related to cracked tooth were also used for the search. A manual search was carried out for selected citations in the located articles to initially select articles.

Article selection criteria are as follows: the article should significantly introduce the concept of cracked teeth; enhance the understanding of the mechanism of cracked teeth; provide true and reliable experimental data or clinical examples; or provide reasonable “expert opinions” in this direction.

## 2. Etiology

A comprehensive understanding of the etiology of CTS is an important prerequisite for its prevention, diagnosis, and management. The predisposing factors for CTS are factors that increase the force acting on the teeth or those that weaken the resistance of the teeth to the chewing force [11] (Table 1). The etiology of CTS is diverse and is related to noniatrogenic and iatrogenic factors that dictate the intervention required. While the former includes developmental and functional status as well as pathological processes, the latter involves the odontiatrogenic factors [28].

### 2.1. Noniatrogenic Factors

#### 2.1.1. Aging

Aging is one of the most important factors in the etiology of CTS. Some studies have shown that CTS mainly occurs in individuals >40 years of age [29]. As age increases, the degree of fatigue of the teeth increases, hard tissues become fragile [12], and the elasticity of the dentin is also lost. Therefore, when the force imposed on the teeth exceeds the limit of dentin elasticity, the teeth crack [13].

#### 2.1.2. Oral Habit

Bad oral habits, such as thermal cycling eating habits [5], long-term unilateral chewing, sleep bruxism, and precocious occlusion, promote CTS [13]. The normal bite force in humans ranges between 3 and 30 kg; hence, when teeth are subjected to forces beyond this range, such as chewing hard objects, the probability of the tooth structure getting damaged increases [5].

#### 2.1.3. Dental Structure

The structural defect in the tooth: deep occlusal and large vertical radicular grooves or bifurcations and even extensive pulp spaces are all tooth structures that can cause CTS. Local areas of the teeth appear structurally weak due to incomplete fusion of the calcified areas during the developmental stages. These local areas may also lead to CTS [12].

Cusp inclination: the tooth anatomy can partly explain the risk indicators for CTS [15], the tip tilt being one of the key parameters. Related studies have shown that the horizontal component of the bite exerts force at the bottom of the fovea and the tooth neck. The tensile stress of the area, thus, increases with the increase in the cusp inclinations. Thus, the high and steep cusp inclinations of the teeth can promote the formation of tooth cracks [16].

### 2.2. Odontiatrogenic Factors

#### 2.2.1. Head and Neck Radiotherapy (HNRT)

**Figure 2:**
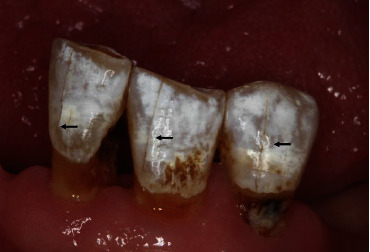
Clinical image of enamel crack lines (arrows) in patients affected by HNRT [35].

HNRT impacts the tooth structure. On one hand, it directly affects the tooth structure, changing the protein composition that forms the cleavage plane of the tooth as well as affecting the production of porous enamel at the dental cervix [17, 30]. Conversely, HNRT leads to synergistic effects of clustering of oral symptoms [18–20]. For example, a decrease in saliva flow will cause the tooth enamel to become brittle in a dry environment; that is, the fracture toughness will decrease, the organic matter in the dentin-enamel junction (DEJ) will get reduced due to dehydration, and the anchorage between the dentin and enamel gets reduced. The altered characteristics of the DEJ would also change the distribution of mechanical stress [31, 32], thereby increasing the probability of enamel craze line (ECL) [33]. In addition to saliva alterations, the daily diet of patients undergoing HNRT tends to lead to low oral pH, resulting in tooth demineralization [34]. This in turn affects the tooth structure and alters its biomechanical properties and, thus, contributes to the high incidence of ECL (Figure 2) [36]. Multiple causes can also have a cumulative effect, thereby increasing the incidence of ECL.

#### 2.2.2. Root Canal Therapy

The incidence of cracks and dentin defects is higher after root canal preparations [23, 37–40]. During this process, the dentin is stressed due to the contact between the instrument and the canal wall, which may lead to the occurrence of CTS [21]. When root canal therapy is performed in a dry environment, even for a short period of time, it may have a harmful effect on the dentin, and the probability of the occurrence of CTS increases [33]. The placement of the intracanal post, use of higher concentration of sodium hypochlorite, and obturation techniques may also contribute to the occurrence of vertical root fractures (VRFs) [22, 41–44]. There is a significant correlation between the formation of CTS and the amount of dentin removed, and excessive widening of the root canal can also increase the risk of VRFs [23].

#### 2.2.3. Restorative Procedures

Restorative procedures can reduce the structural strength of teeth as well as increase the risk of cracked teeth by almost 29 times [45]. Intracoronal restorations can further lead to the occurrence of cracked teeth by promoting a sharp inner line angle and the old caries design, which weaken the structural strength of teeth and cause stress concentration [24]. Many restoration procedures, such as the placement of “friction locks” or “self-threaded dentin pins,” may cause stress on the residual tooth structure and eventually result in cracks. Large amalgam restorations of the mandibular molars are often accompanied by tooth cracks [46]. When a poor-quality amalgam restoration is placed or when moisture and excessive condensation pressure contaminate the newly placed amalgam during restoration, it may also lead to CTS [41, 47, 48]. Other aspects of restorative procedures, such as excessive removal of tooth tissue during the preparation of the tooth cavity, have been shown to significantly reduce the hardness of the tooth and may also lead to the formation of cracked teeth [25].

#### 2.2.4. Material Performance

Compared to nonmetallic materials, metallic materials are more likely to cause CTS [26]. There is a difference in the thermal expansion coefficient between the tooth and the restoration material, and this may also cause CTS [27]. The restoration material deforms under the action of an external force or the influence of the oral environment, thereby causing abnormal bite force distribution which easily leads to CTS. Therefore, the properties of the material being used are of great significance in preventing the occurrence of cracked teeth.

#### 2.2.5. Stress Function

Guersten [49] found that the excessive force exerted on healthy teeth or the weakened physiological forces of teeth can cause incomplete fracture of the enamel or dentin. Moreover, the stress exerted on the teeth during the extraction process can have an impact on the formation of cracks, and tooth slices are also a cause of dentin crack formation [23].

## 3. Diagnosis

During earlier stages of CTS, when the symptoms and signs are frequently blurred, it is generally difficult for dentists to diagnose cracked teeth. Dentists must arrive at a clear diagnosis as much as possible [48]. Currently, there are multiple techniques that include clinical examination, auxiliary iconography examination, and a few new types of technologies toward the diagnosis of CTS.

### 3.1. Clinical Examination

**Figure 3:**
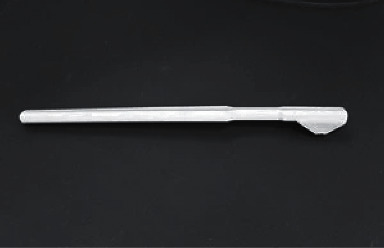
Tooth Slooth tool for the bite test [13].

When the patient has clinical symptoms, the dentist can use a combination of the percussion, point load, and cold stimulation test to find the sick tooth [50] or use a special plastic bite block (for example, Tooth Slooth, Professional Results Inc.; Figure 3) for the bite test [51]. However, some researchers believe that applying pressure to teeth with suspicious symptoms may result in further propagation of cracks and, due to the associated risk, do not recommended point load and bite tests [52].

Methylene blue dye staining assists the visual detection of coronal cracks. This is due to the aggregation tendency of the dye. However, the use of the dye may hide cracks or lead to subtle color changes in the deeper layers of the enamel [53]. Furthermore, the original restoration materials need to be removed before applying the dye which takes 2–5 days [54].

Fiber-optic transillumination (FOTI) is a cross illumination of an optical fiber probe placed at different points on the surface of the crown or root. Because the refractive index of the crack differs from that of the peripheral tissue, there is a back reflection of the light reaching the crack resulting in the fracture line being distinctly displayed [55]. FOTI not only assists clinicians in enhancing the rate of clinical diagnosis but also serves as an appropriate machinery for adjuvant therapy, such as lighting the root canal orifice during root canal therapy [56]. An operating microscope is a commonly used equipment for the assisted diagnosis of CTS [53]. Dentists usually diagnose cracks by observing the peripheral crack lines of the fractured surface. However, the observed crack lines do not represent their size and shape [57].

Experienced clinicians have suggested that the magnification used to assess the range of enamel cracks is approximately 14–18 times, and 16 times is the optimal magnification used for evaluating enamel cracks [53]. Structural and smaller cracks can be appropriately distinguished or observed by using the FOTI equipment and the dental operating microscope. They are well-received and valuable diagnostic accessories for CTS and dental caries and are extensively used in clinical practice.

### 3.2. Radiography

While the traditional periapical X-rays (PR) can only provide a definitive diagnosis when the deviation of the root fracture is obvious [58], cone-beam computed tomography (CBCT) can detect subtle loss of the periapical bone during VRFs [59, 60]. However, the resolution of CBCT being only approximately 80 *μ*m, it is not suitable for the clinical diagnosis of cracked teeth and detecting early VRFs [61, 62].

In an *in vitro* study, Yuan et al. [63] demonstrated that compared to the conventional approach, scanning using CBCT can be enhanced using meglumine diatrizoate as a contrast agent as it can objectively and effectively show hidden cracks. Therefore, some researchers consider this an auxiliary method in the imaging of the tooth periapex [64].

### 3.3. New Technology

#### 3.3.1. Swept-Source Optical Coherence Tomography (SS-OCT)

**Figure 4:**
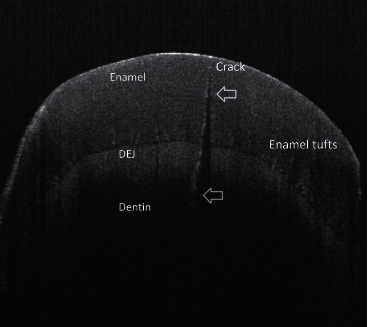
SS-OCT image of the dentin crack. The crack extended beyond the DEJ and displayed as a bright white line [68].

SS-OCT is a promising technique for the detection and analysis of incipient enamel caries and early CTS. SS-OCT is a variant of the fast Fourier transform algorithm, which emits different wavelengths of light using a laser source with variable wavelengths (at the near-infrared wavelength of 1300 nm, the enamel and cracks have high transparency and high contrast, respectively) [65]. This is a nondestructive imaging technique that uses low-coherence interferometry to detect the reflection signals of biological tissues at different depths facing the incident weak coherent light and achieves two- or three-dimensional structural images through a laser scanner and semiconductor camera [66, 67] (Figure 4). A study by Lee et al. [69] showed that the diagnostic accuracy of SS-OCT is better than that of micro-CT, FOTI, and visual inspection [70]. Although SS-OCT has the advantage of enhancing the resolution, the specificity of SS-OCT in detecting full-thickness cracks is weak because the enhanced image of deep enamel cracks often overlaps with the enamel plexus. SS-OCT has a confined penetration depth in the coronal part within 3 mm that can be irradiated by laser. Hence, its main application is restricted, and it is suitable only for early diagnosis [69, 70].

#### 3.3.2. Near-Infrared Imaging

Recently, Li et al. [71] confirmed the practicability of using indocyanine green-assisted near-infrared fluorescence (ICG-NIRF) imaging to detect enamel-dentin and enamel cracks *in vitro*. ICG-NIRF imaging can detect more cracks than using only the second region of near-infrared light, which detects the cracks and their depths not detected by CT and X-ray. But, it cannot distinguish crack types and obtain accurate crack depth information.

### 3.4. Other Techniques

#### 3.4.1. Ultrasonic System

Detection of CTS using the ultrasonic system has a promising future because it has the ability to penetrate hard tissue (in theory, penetrating some radiopaque restorations) and lacks the hazards associated with ionizing radiation. Culjat et al. [72] successfully applied an integral ultrasonic system to detect known cracks in simulated teeth. Additionally, Sun et al. [73] were the first to employ a laser ultrasonic system that combined analysis of scanned images with a finite-element method to precisely detect the depth for detecting human CTS in clinical settings.

#### 3.4.2. Infrared Thermography

The application of infrared thermography technology can assist detecting small cracks (4–35.5 *μ*m), when other diagnostic techniques fail. Because the smaller cracks will be vibrated by ultrasonic power (the amplitude and detection angle should be 0.89 W and within 45°, respectively), local friction that occurs under the effect of vibration consequently generates heat [74, 75]. The microcracks of the dentin can then be displayed under the action of the thermal imager. Nevertheless, this method has significant limitations in detecting wide cracks [76].

#### 3.4.3. Near-Infrared 810 nm Diode Laser

A near-infrared 810 nm diode laser can be used as a new technology to assist in the management of symptomatic CTS. When the laser energy is irradiated on teeth with suspicious symptoms, most patients experience sharp pain, and only few of them get dull pain. This may be because when the laser beam enters the depth of the crack, the energy that is applied to the pulp causes an analogous irritation [8].

In clinical diagnosis, the commonly used diagnostic methods and new technologies need to pay attention to problems arising in the earlier process.  Table 2 provides a summary of the available technologies.

## 4. Management

As dentists have gained more knowledge on CTS, several approaches are being used toward its management, each having its own advantages and disadvantages. These have been summarized in Table 3. The management plan is different in different departments. In some severe conditions such as the occurrence of irreversible pulpitis and excessive extension of cracks, accordingly, root canal therapy and extraction may be required [92].

### 4.1. Immediate Management

#### 4.1.1. Occlusal Adjustment

From the perspective of many authors, once dentists diagnose CTS (especially when cracks are on mandibular lingual cusps), occlusion is adjusted to minimize the loading on the CTS tooth, thereby alleviating the symptoms and delaying the cracking process. After occlusal adjustment, the restoration should be placed on the affected cusp to avoid any further fractures in the tooth [47, 82].

#### 4.1.2. Copper Rings and Stainless Steel Bands

Copper rings and stainless steel bands can be used for the diagnosis and immediate management of early CTS cases, whose cracks are observed extending beneath the gingival margin or in which one or more surfaces of the tooth are missing [93, 94].

As they act like a splint, the incorrect diagnosis of CTS is avoided. If the pain does not subside following the use of them, it indicates that the diagnosis may be incorrect or further endodontic treatment may be required [51]. In cases where symptoms resolve with the use of a tight stainless steel band, a full crown may be used to replace the previously placed restoration. Following splint fixation, occlusal tests must be repeated to confirm the diagnosis [49]. Stainless steel bands are preferable to copper ones because their contoured shape results in low gingival irritation. The drawbacks of stainless steel bands include the requirements of prefabricated belts and their large inventory. However, this can be solved by quickly processing the bands using forming pliers and spot welding [51, 93]. Nevertheless, they may not be used in dental management in most cases because many modern dentists lack the experience of placing them. Moreover, the rings must be produced in advance [95].

#### 4.1.3. Direct Composite Splint (DCS)

The DCS was developed based on the concept of “Dahl” [96]. Except in cases with limited eruptive potential, this occlusal contact was reconstituted after a period of time by a combinatorial procedure of alveolar segment intrusion and extrusion [95].

**Figure 5:**
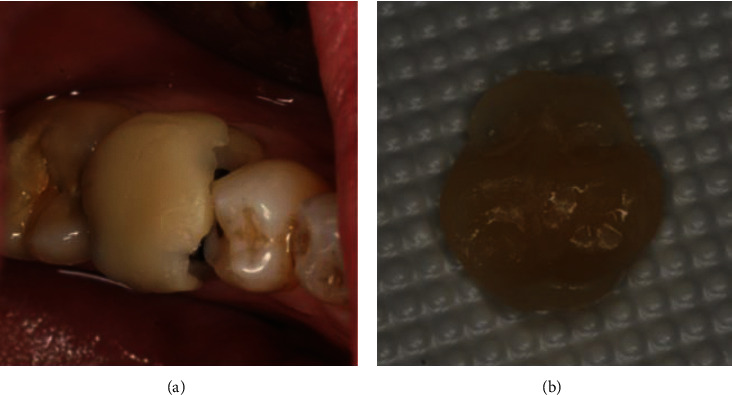
A DCS restoration utilized as a diagnostic aid: (a) upper view of the restoration; (b) lower view of the restoration [83].

The DCS does not require any prior tooth preparation and has the advantage of easy removal, and though it is marginally invasive, it can be used for short-term management. It can be applied quickly and easily because its application does not require details of anatomical contours, and materials and equipment for its fabrication are readily available (Figure 5). However, adherence to careful inclusion criteria, selection of a suitable composite resin, the application method, and the use of proper bonding systems need to be considered [83].

#### 4.1.4. Temporary Crown

A crack is fixed after the placement of the full-coverage crown. The occlusal forces are diverted and dispersed over the entire prepared tooth surface, thus minimizing the pressure on the cracked area. The crown is held in place by friction, and surface cementation prevents the movement of cracks during mastication. However, the fabrication of a temporary crown for the interim management of an incomplete fracture of a posterior tooth is time consuming and intrusive, and it is extremely cumbersome to remove the posterior tooth [97]. Additionally, as tooth preparation and temporary crown fabrication take some time, it may delay the fixation of the fracture, resulting in its continued expansion. Due to continued bleeding, the risk of endodontic complications increases [98].

### 4.2. Direct Restoration

Clinically, patients rarely visit a doctor with a dental crack as a complaint because patients with slight dental cracks generally have no discomfort, despite the appearance of an asymptomatic fracture line on the tooth enamel. Moreover, patients' oral health awareness being weak, the chances of early detection and direct repair are minimal. Generally, direct repairs are usually performed by dentists when patients visit them for undergoing treatment for dental caries and other oral diseases [99].

Compared to the use of direct onlays, occlusal overlay restorations in the management of CTS may not be particularly skill demanding. The height of the cusp should be reduced during the restoration procedure to reduce the risk of fracture due to lateral loading. If the cracks are very fine in the early stage, we can closely observe or/and use composite resin to fill them [100]. When the fracture surface is completely above the gingiva or no more than 1–3 mm, a thin drill can be used along the crack line until the tooth appears in a healthy and normal structural state, to directly repair the tooth structure. There is no significant reduction in the structural stability of the teeth during thin drilling because tooth structures along the fracture surface are not chemically bonded [57].

Direct and indirect composite resin restorations fully protect the cracked teeth, but direct restorations have a higher survival rate when the load force is over 1000 N [90].

Bonding restorations can effectively restore the strength of teeth weakened by cavity preparation, and Staninec and Holt [47] demonstrated that amalgam can be cemented to the etched enamel and dentin with an adhesive liner. However, when some studies evaluated the efficacy of direct composite intracoronal resin restorations (including amalgam restorations) for the management of painful and cracked posterior teeth over a 7-year follow-up period, they found that bonded silver amalgam restorations were less favorable than resin-covered restorations that received direct bonding. This may be because, in the coronal approach, the adhesive interface between the tooth and the restoration gets progressively damaged under periodic functional loads [52].

Compared with unrepaired teeth, amalgam restorations show a significant increase in fracture resistance. Composite resin restorations are more resistant to fracture than amalgam restorations, and the strength of premolars restored with composite resin restorations has been reported to be approximately twice that of the unrepaired premolars [101, 102].

Age is also a factor in the selection and use of repair materials [103, 104]. Composite and amalgam restorations also differ in the incidence of cusp fractures among patients depending on their age. Although there was no significant difference in the incidence of apical fractures between amalgam and composite restorations in younger patients (18–54 years), in older patients (55–96 years), the incidence with composite restorations was higher than that with amalgam restorations. With increasing age, the basic metabolic rate of the tooth tissue (especially dentin) decreases and is accompanied by continuous tissue dehydration and loss. Due to this, the brittleness increases, and thus, it is prone to fracture. Gradually, oral cracks form and expand to the surroundings. However, regardless of the material used, temporary restorations do not maintain the same resistance to chewing pressure as against the restorations performed with stronger dental material [105].

### 4.3. Indirect Restoration

#### 4.3.1. Inlay Restorations

Practitioners acknowledge that inlay restorations undermine the residual tooth structure when preparing the tooth, resulting in tooth fractures occurring on unprotected surfaces [88]. Furthermore, traditional inlay restorations use a “wedge retention” concept, which can create periodic occlusal pressure on the tooth before bonding and during use. Therefore, conventional inlays are ineffective in the management of CTS cases [95, 106].

The effects of inlays with adhesive materials are quite different. An *in vitro* experiment demonstrated that indirect resin-bonded composite inlays, as well as bonded mesial-occlusal-distal (MOD) ceramic inlays, have the ability to improve the fracture strength of prepared teeth to a level similar to that of healthy teeth [107].

Compared to ceramic inlays, resin composite inlays experience greater wear in the early stages at the bonding site, but there is no difference between the two in terms of long-term usage. This may be due to the different locations of stress distribution on the affected teeth covered with the two different material restorations. The stresses on ceramic inlays are mainly distributed on the ceramic structure. However, resin composite inlays release shrinkage stresses at the interface of the tooth structure thereby promoting the creation and extension of cracks in the enamel. Therefore, ceramic inlays can be more efficient in restoring CTS than resin composite inlays [108].

The advancement of computer-aided design/computer-aided manufacturing (CAD/CAM) technology is worth mentioning. A study showed that CAD/CAM-fabricated resin inlays improved accelerated fatigue resistance and reduced the propensity for cracking in large MOD restorations compared to direct resin restorations [109].

#### 4.3.2. Onlay Restorations

**Figure 6:**
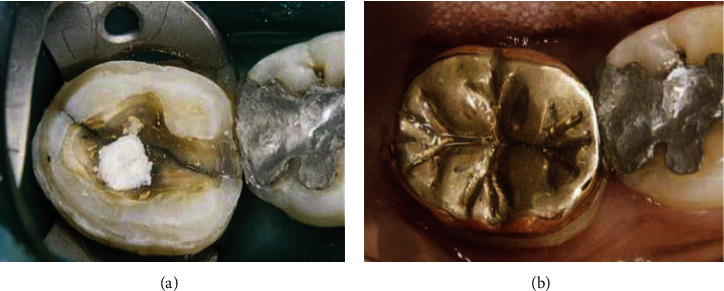
Restoration of a fractured right mandibular second molar using a gold onlay. (a) A cracked tooth prepared for restoration via onlay bonded with resin; (b) 22 months after restoration via bonded type III gold alloy inlays with Panavia Ex cement [110].

Brackett et al. [89] referred to gold onlays as the most conservative restoration approach. In this study, gold onlays were cemented with resin-modified glass ionomer luting cement (Figure 6). Annual examinations over the next six years showed a solid, defect-free onlay with no symptomatic recurrence [89]. A clinical review also concluded that the survival rate of type III gold alloy (Firmilay, Jelenko, San Diego, CA, USA) restorations bonded with Panavia Ex cement (Kuraray Co., Japan) at 60 months was 89%, which is satisfactory [110]. Microscopic mechanical cementation methods, such as using composite materials and resin-modified glass ionomers, are emerging, permitting alloy restorative preparation methods to evolve toward simpler directions [110].

Ceramic onlays have the advantages of excellent properties such as resistance to wear and friction, outstanding appearance, and biocompatibility. Compared with resin prostheses, it can restrict defects due to polymerization shrinkage due to cement particles. This property in turn helps to maintain the adhesive capability of cement, subsequently increasing the stability as well as improving the fracture resistance after repair to a certain extent. Ceramic inlays are also considered an effective method for preserving the tooth structure due to reduction in the loss of the hard tooth tissue [84].

A study of the long-term use of lithium disilicate ceramics showed that, after 11 years of use, the restorations showed only a small amount of discoloration and one fissure [111]. All restorations were preserved without complications. However, the margin shape may influence the inlay survival rate.

Indirect composite resin onlay restorations have been proven to be effective in treating painful, cryptically fractured teeth, both in *in vitro* studies and in clinical practice [7, 112]. They also have a higher fracture resistance than ceramic onlays [112]. The survival ratio of indirect composite resin onlays did not differ significantly from that of direct composite resin restorations. The higher level of polymerization conversion of indirect composite resin onlays leads to better mechanical and physical properties compared to those of direct composite resin, and the cavity design resulted in lower fatigue resistance, higher cost, and more invasiveness [90].

#### 4.3.3. Full Crown

Full crowns can be the first choice of treatment, both with and without the symptoms of CTS [113]. Gutherie and DiFiore claimed [97] that full-coverage crowns best meet the target of treating CTS. Especially after root canal therapy, the survival rate of cracked teeth restored with a full crown is significantly higher than that restored with others, and the incidence of complications is reduced [91, 94, 114]. With the usage of acrylic resin crowns for the treatment of CTS, the failure rate was 11%.

To meet the esthetic requirements of patients, metal-ceramic crowns (CMCs) are frequently used as fixed restorations. In a retrospective study by Cheung et al. [51], the estimated 10-year survival rate of pulpal activity for CMCs was 84.4%. However, pulpal injuries may arise at the time of crown placement, which may require consequential root canal therapy. Therefore, regular radiological follow-up is necessary [85].

## 5. Prevention

Prevention plays an important role in halting the occurrence and development of CTS caused by medical, environmental, or genetic factors.

Individuals should maintain good oral hygiene by adopting good cleaning practices, developing healthy chewing habits, and following a proper diet (such as avoiding clenching, extensive grinding, abrasion, bruxism, eating betel nut, and hard food). Additionally, increasing the frequency of oral examinations can also be effective in preventing CTS, which is especially important for the elderly.

When patients are treated for caries or other periapical diseases, medical workers should avoid secondary damage or secondary crack generation to the affected tooth and surrounding teeth as much as possible. Dental appliances such as hard acrylic and soft splints can prevent CTS by dividing the force throughout the masticatory system and decreasing the frequency, but not the intensity of bruxism. Appliances should be worn continuously because once the appliance is removed, the muscle activity may return to previous levels [115].

When symptoms of CTS occur, occlusal adjustments or bonded restorations can be performed to prevent further extension of the cracked tooth [82].

## 6. Conclusions

CTS is a common, multiple, clinically significant tooth fracture caused by a variety of factors.

From the common clinical diagnosis methods to the newer techniques represented by SS-OCT, the diagnostic accuracy of CTS has been advancing continuously. In clinical practice, the choice of management options by dentists is not uniform, and the majority of doctors recommend full crown treatment. To summarize, with the development of clinical techniques, it is believed that the occurrence of CTS will become more predictable, diagnosable, and amenable to management.

## Figures and Tables

**Table 1:** Etiological factors of cracked tooth syndrome.

Etiological factors		
Noniatrogenic factors	Aging	Increasing levels of dental fatigue [12]
		Weakened dental hard tissues [12]
		Lost dentin elasticity [13]
		Increasing number of restored teeth [14]
	Oral habit	Thermal cycling eating habits [5]
		Long-term unilateral chewing [13]
		Sleep bruxism [13]
		Precocious occlusion [13]
	Dental structure	The structural defect in the tooth [12]
		Cusp inclination [15, 16]

Odontiatrogenic factors	HNRT	Affects the tooth structure [17]
		Synergistic effects of clustering of oral symptoms [18–20]
	Root canal therapy	The contact between the instrument and canal wall [21]
		Use of higher concentration of sodium hypochlorite [22]
		Excessive widening of the root canal [23]
	Restorative procedures	Stress concentration [24]
		Excessive removal of tooth tissue [25]
	Material performance	Metallic materials [26]
		Difference in the thermal expansion coefficient [27]
		Material deformation
	Stress function	

**Table 2:** of four common techniques in detecting cracked teeth.

Features	Transillumination	Intraoral X-ray	CBCT	SS-OCT
Distinguish the type of crack	**×** [55]	**×** [74]	**×** [77]	**○** [70]
Show root fractures	**×** [55]	**○** [48]	**○** [63]	**×** [70]
Determine the crack depth	**×** [77]	**×** [78]	**○** [79]	**○** [68]
Produce radiation	**×** [56]	**○** [80]	**○** [81]	**×** [66]

**○**, meet the description; **×**, does not meet the description.

**Table 3:** Therapy methods and, accordingly, their advantages, disadvantages, and comparisons.

Therapy method		Advantage	Disadvantage	Comparison
Immediate treatment	Occlusal adjustment	Alleviating the symptoms Delaying the cracking process [82]	Weakens the natural tooth structure [82]	(1) Copper rings and stainless steel have the highest technical sensitivity (2) DCS is the most minimally invasive of the four techniques (3) Temporary crowns are more prone to delayed treatment
	Copper rings and stainless steel	Helping with the establishment of definitive diagnosis [13, 51]	Skills and knowledge are required Food trapping [57]	
	Direct composite splints (DCSs)	Easily removed [83]	Careful inclusion criteria [83]	
	Temporary crown	Aesthetical and practicable advantages [84] Most practitioners are competent with techniques	Risks of pulp tissue trauma [85, 86]	

Direct restoration		Avoiding provisional restorations and low cost	Risk of fracture due to lateral load [57]	(1) Direct restoration is more minimally invasive than indirect restoration (2) The survival rate of direct restoration is higher than that of indirect restoration when the load exceeds a certain range [90]
Indirect restoration	Inlay restorations	Improving the fracture strength of prepared teeth [87]	Preparation undermines the residual tooth structure [88]	
	Onlay restorations	Conservative [89]	Lower fatigue resistance [90]	
	Full crown	Decreasing the incidence of complications [91]	Pulpal injuries [85]	

## Data Availability

All data, figures, and tables in this review article are labeled with references.
